# Supplementary material for: Novel Plant-Associated Brevibacillus and Lysinibacillus Genomospecies Harbor a Rich Biosynthetic Potential of Antimicrobial Compounds
Source: Microorganisms. 2023 Jan 9;11(1):168. doi: 10.3390/microorganisms11010168 (PMC9867215; doi:10.3390/microorganisms11010168)
Supplement: Supplementary file 1 [file microorganisms-11-00168-s001.zip › Supplemental Figures 03-01-23.pdf]

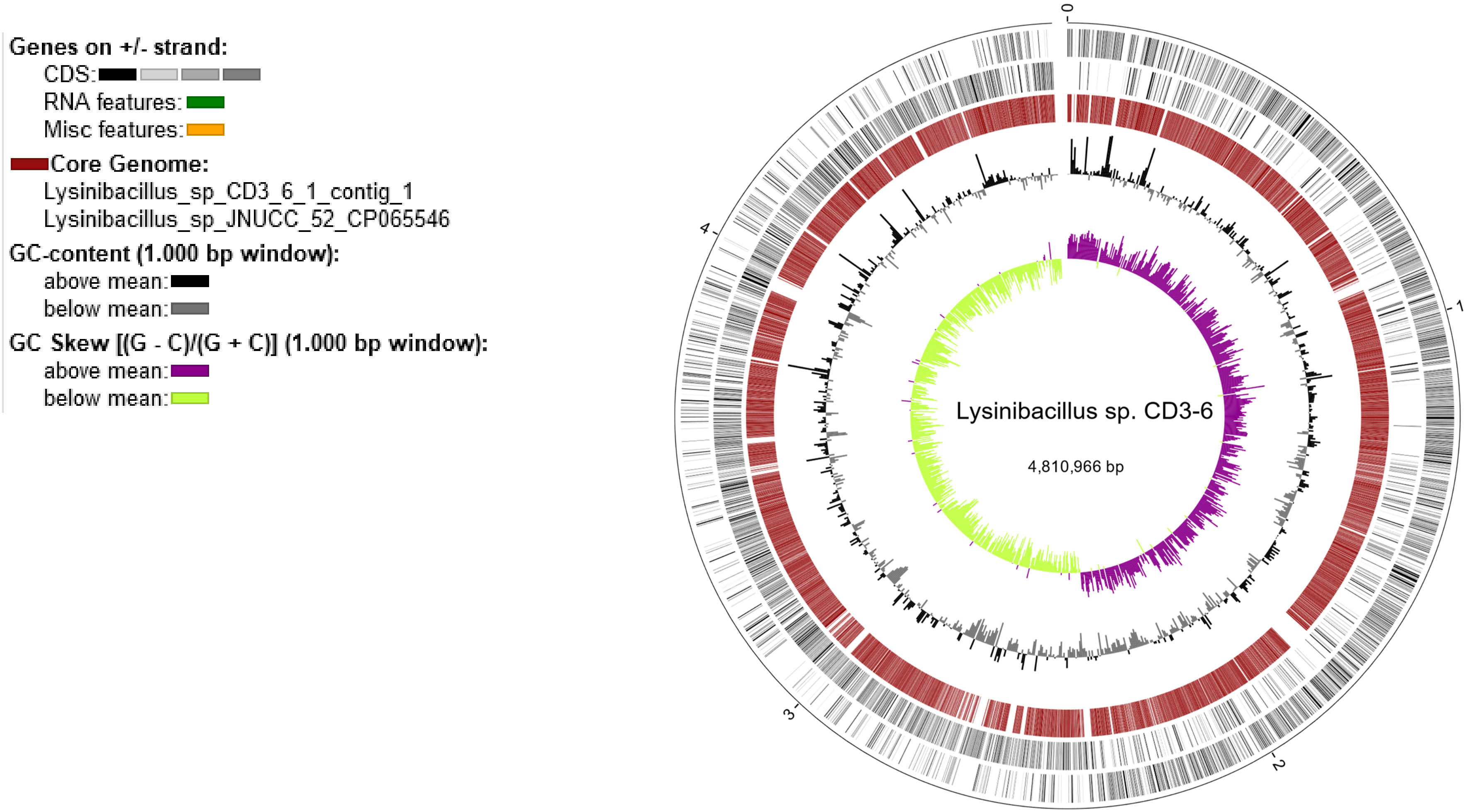


Supplementary Figure S1: Circular plot of the genome of *Lysinibacillus* sp. CD3-6 generated with BioCircos (Cui et al. 2016). The genome of CD3-6 was computed against *Lysinibacillus* sp. JNUCCC-52. Core genome genes shared by Lysinibacillus sp. CD3-6 and Lysinibacillus sp. JNUCC-52 are indicated in brown color in the third circle.

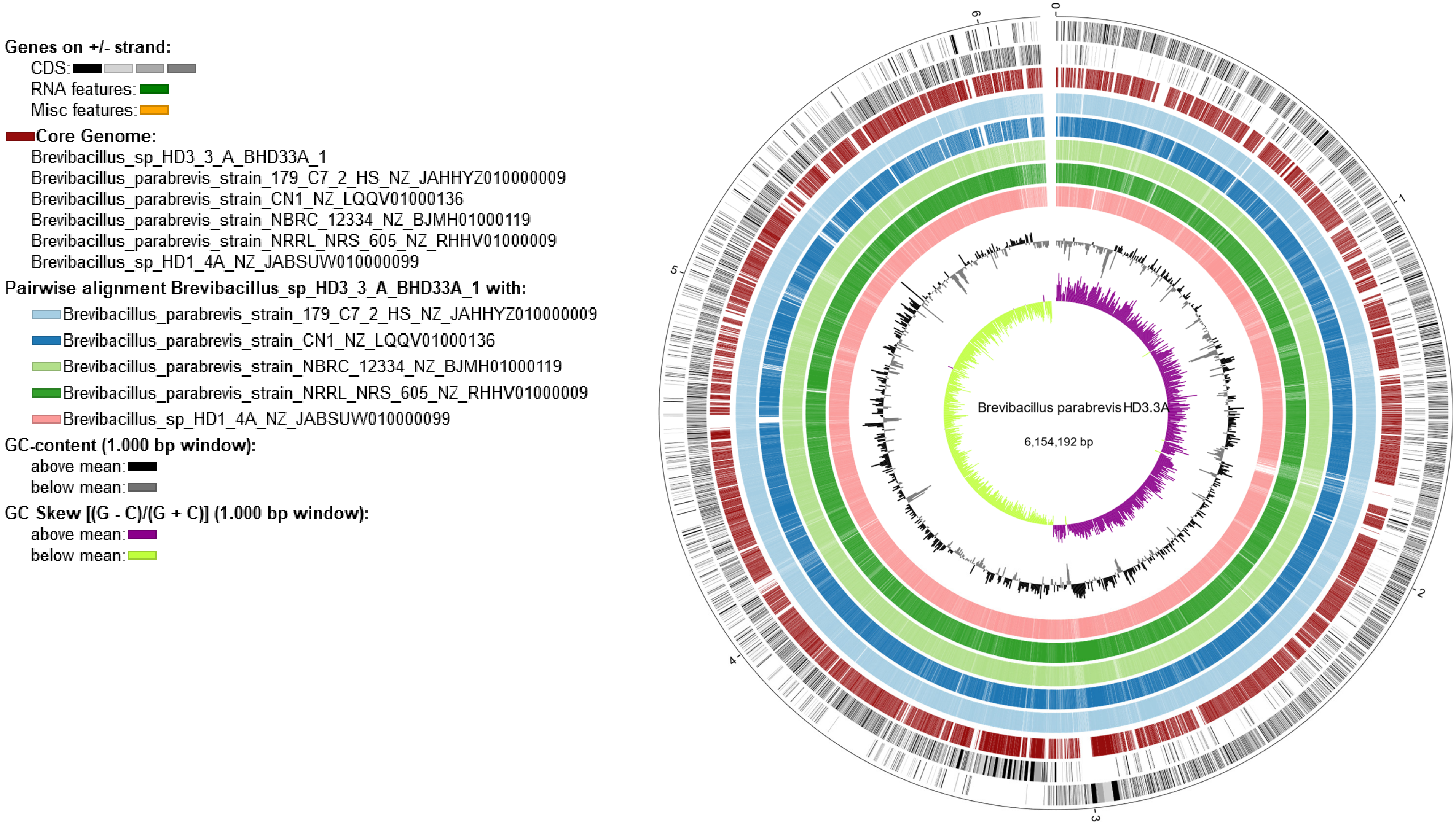


Supplementary Figure S2: Circular plot of the genome of *Brevibacillus parabrevis* HD3.3A generated with BioCircos (Cui et al. 2016). HD3.3A was used as reference for computing the core genome against representatives of the *Brevibacillus*-A5 branch (clusters 25-27, see section 3.2). Genes of the core genome are presented in the third circle.

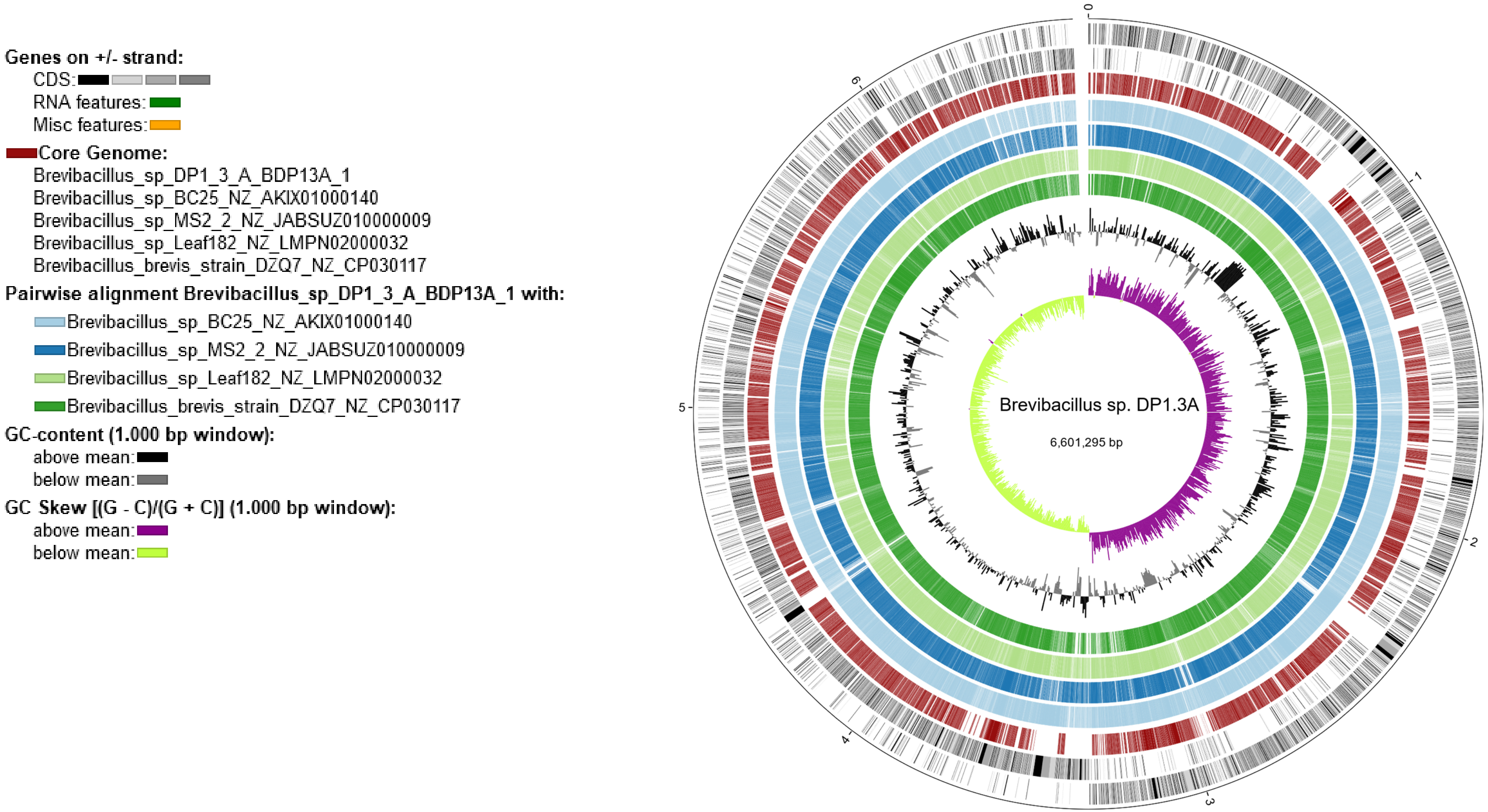


Supplementary Figure S3: Circular plot of the genome of *Brevibacillus* sp. DP1.3A generated with BioCircos (Cui et al. 2016). The DP1.3A genome was computed against selected members of the *Brevibacillus*-A6 branch (clusters 28-35). Homologous regions representing the core genome are visualized in the third circle.

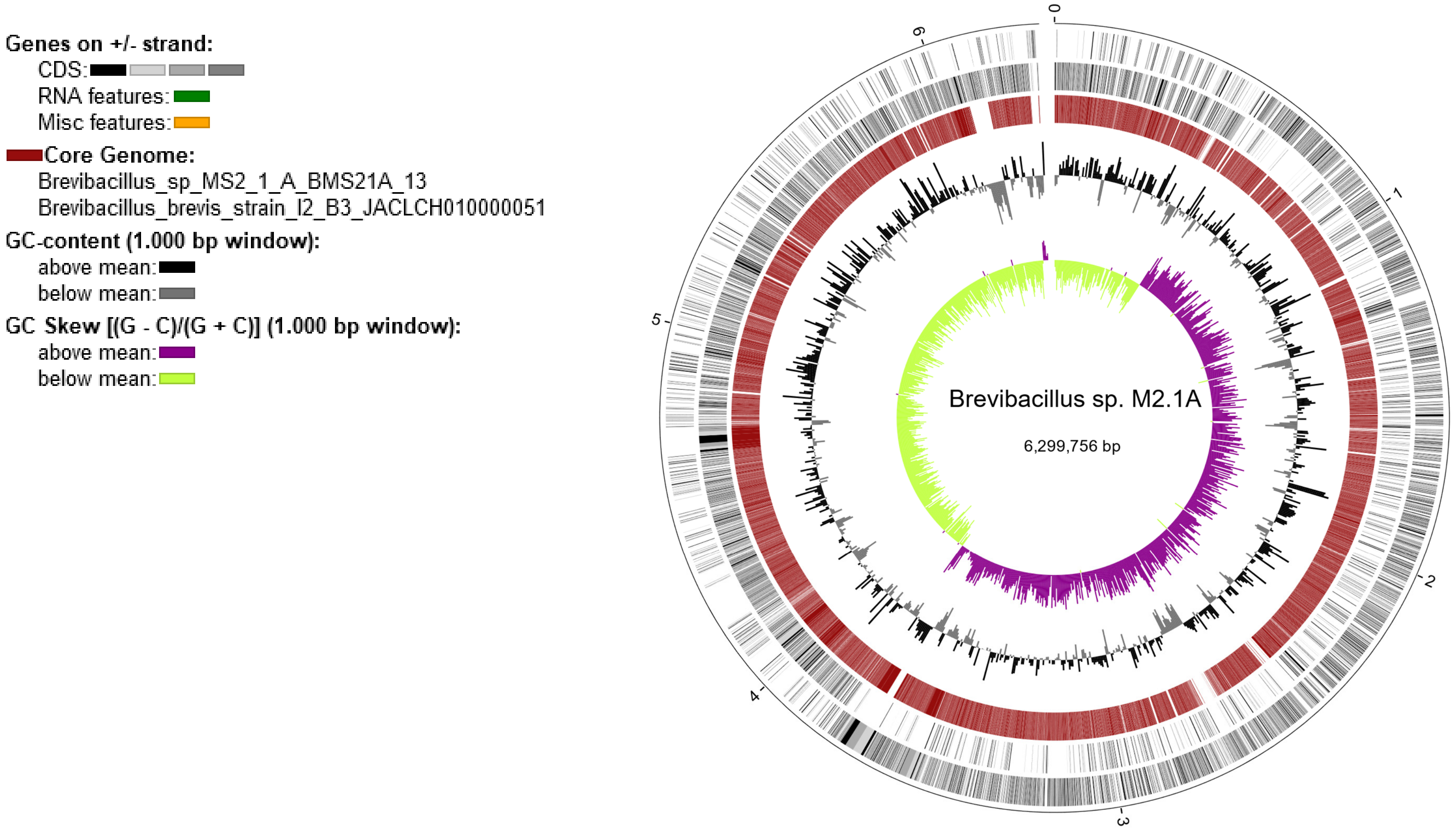


Supplementary Figure S4: Circular plot of the genome of *Brevibacillus* sp. M2.1A generated with BioCircos. The genome of M2.1A was computed against *B. brevis* 12B3. Core genome genes are indicated in brown color in the third circle

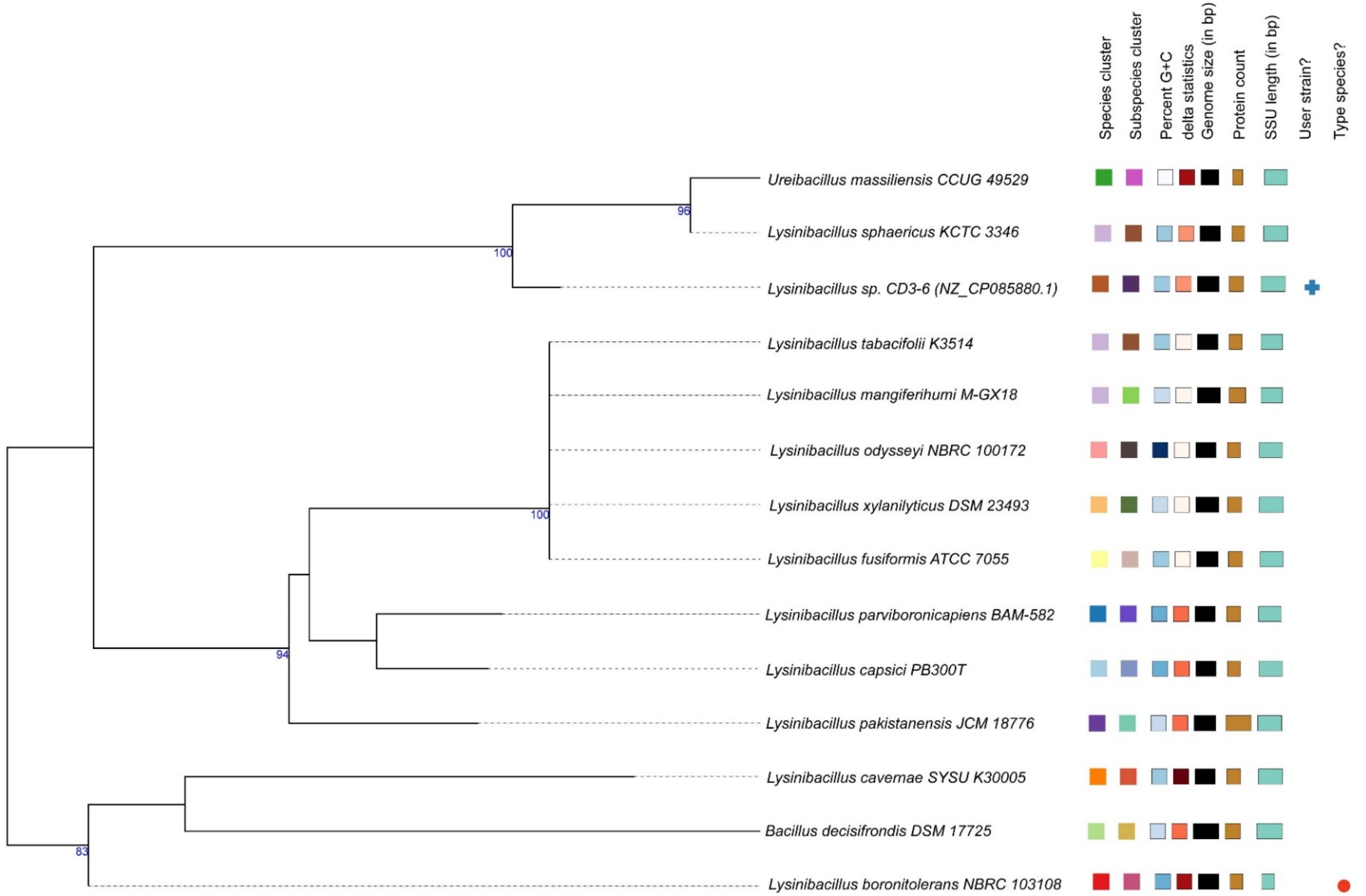


Supplementary Figure S5: GBDP tree (16S rDNA gene sequence based) with *Lysinibacillus* sp. CD3-6. Tree inferred with FastME 2.1.6.1 from GBDP distances calculated from 16S rDNA gene sequences. The branch lengths are scaled in terms of GBDP distance formula $d_5$. The numbers above branches are GBDP pseudo-bootstrap support values > 60 % from 100 replications, with an average branch support of 54.8 %. The tree was rooted at the midpoint.

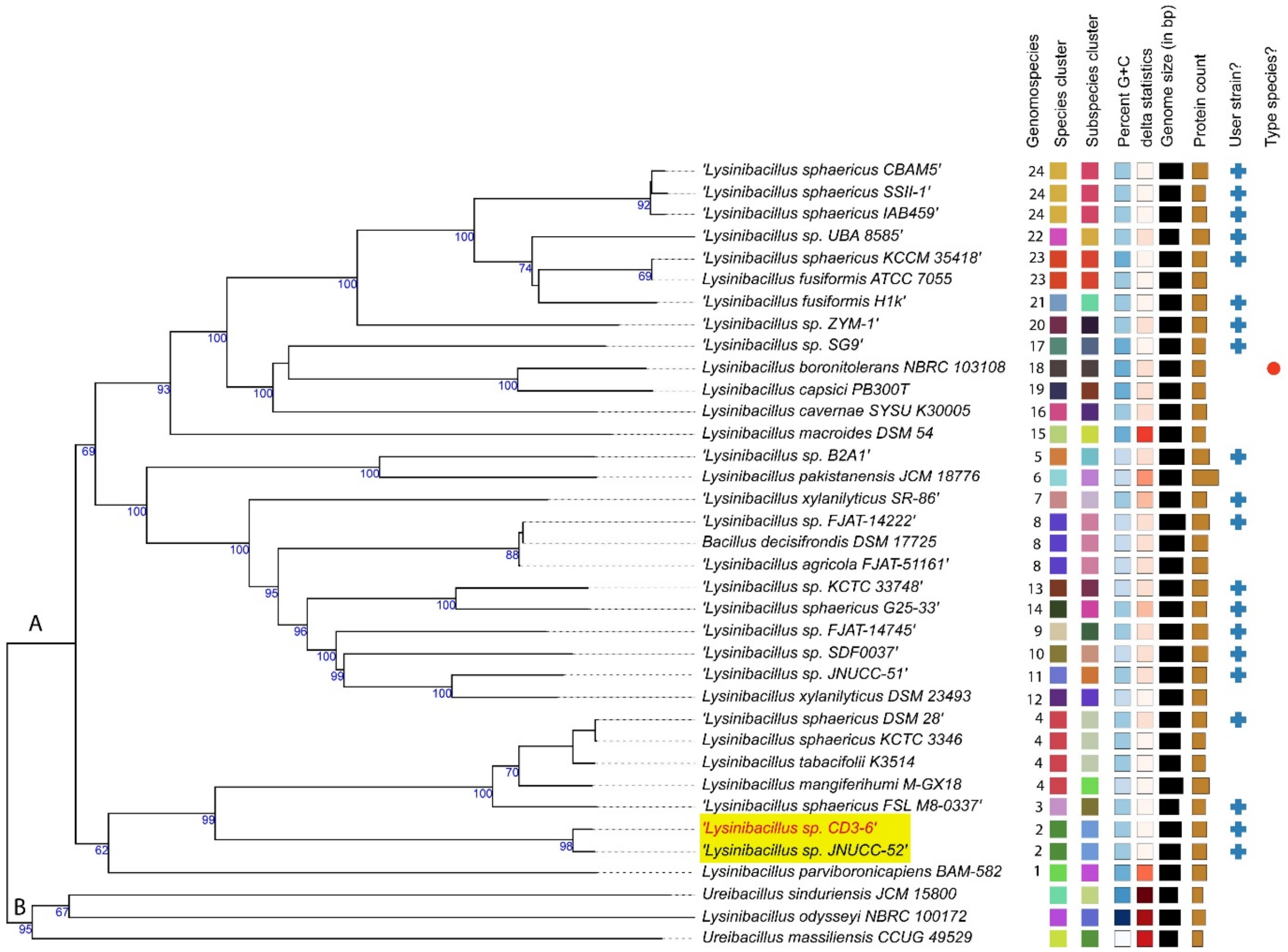


**Supplementary Figure S6:** *Lysinibacillus* tree inferred with FastME 2.1.6.1 from GBDP distances calculated from 37 entries. Clustering of the *Lysinibacillus* strains revealed 28 species and 29 subspecies clusters. Two of them could not been assigned to known species. *Lysinibacillus* CD3-6 was assigned to form together with *Lysinibacillus sp.* JNUCC-52 a novel species cluster. The branch lengths are scaled in terms of GBDP distance formula $d_5$. The numbers above branches are GBDP pseudo-bootstrap support values > 60 % from 100 replications, with an average branch support of 93.5 %. The tree was rooted at the midpoint.

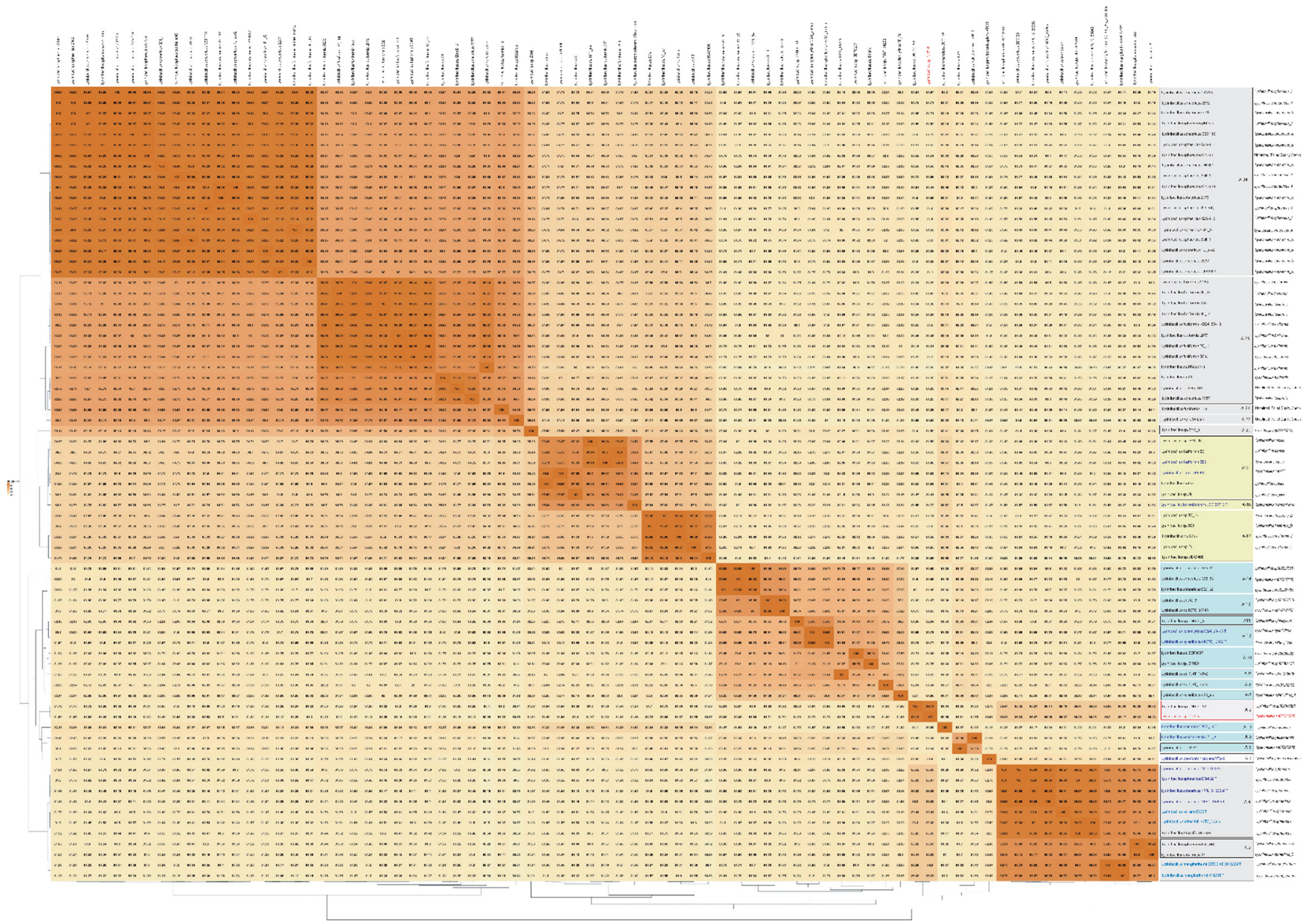

**Supplementary Figure S7**. Heat Map of the FastANI-Matrix [23] of group A *Lysinibacillus* genomes. *Lysinibacillus* sp. CD3-6 (labelled in red letters) isolated from Vietnamese crop plants formed together with JNUCC-52 a separate genomospecies. Average nucleotide identity and aligned nucleotides [%] were determined using JSpecies https://jspecies.ribohost.com/ [42]. For better resolution use the separate png file of Suppl. Fig. S7.

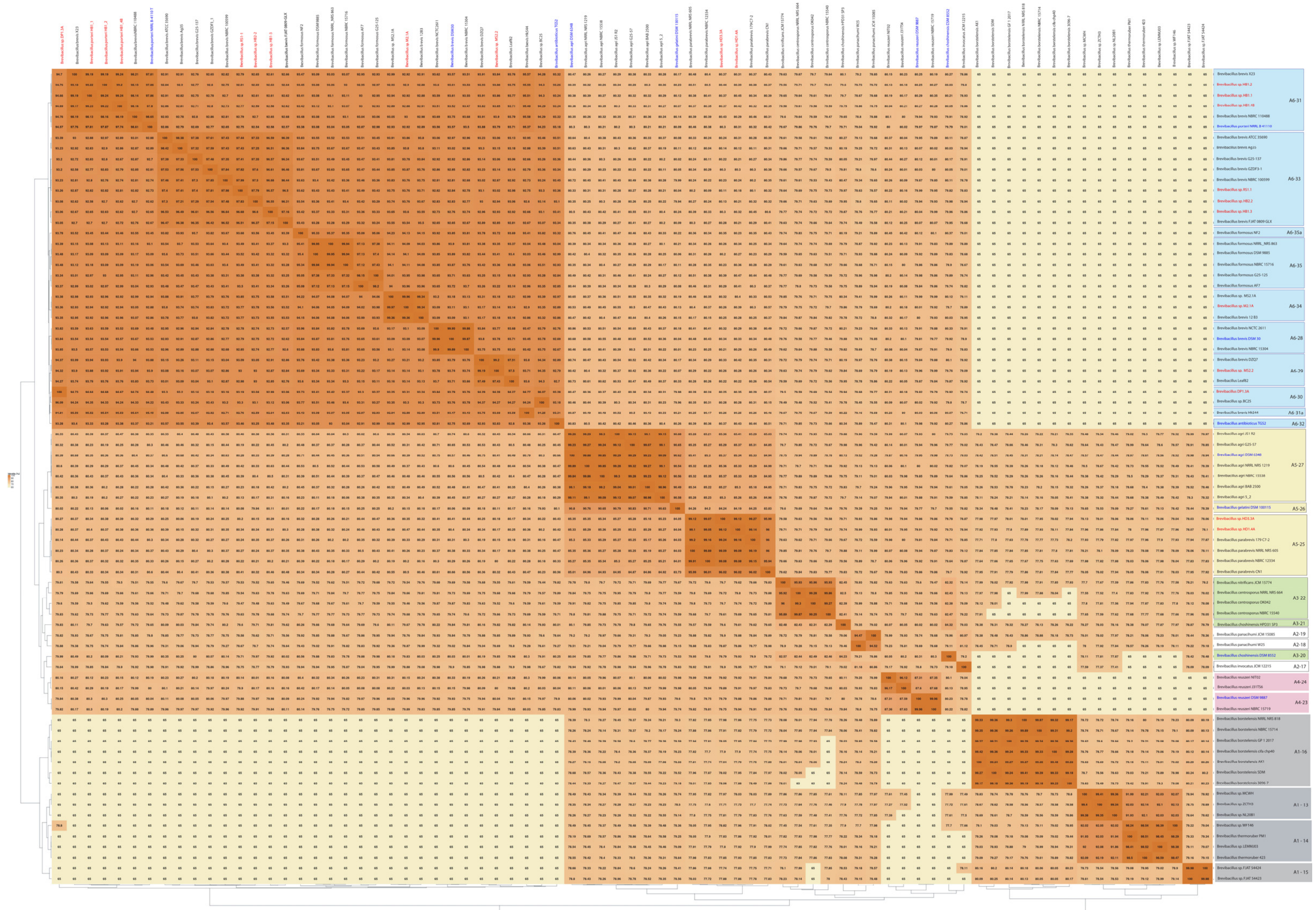

**Supplementary Figure S8.** Heat Map of the FastANI-Matrix [23] of Brevibacillus strains isolated from Vietnamese crop plants. Average nucleotide identity and aligned nucleotides [%] were determined using JSpecies https://jspecies.ribohost.com/ [42]. For better resolution use the separate png file of Suppl. Fig. S8.

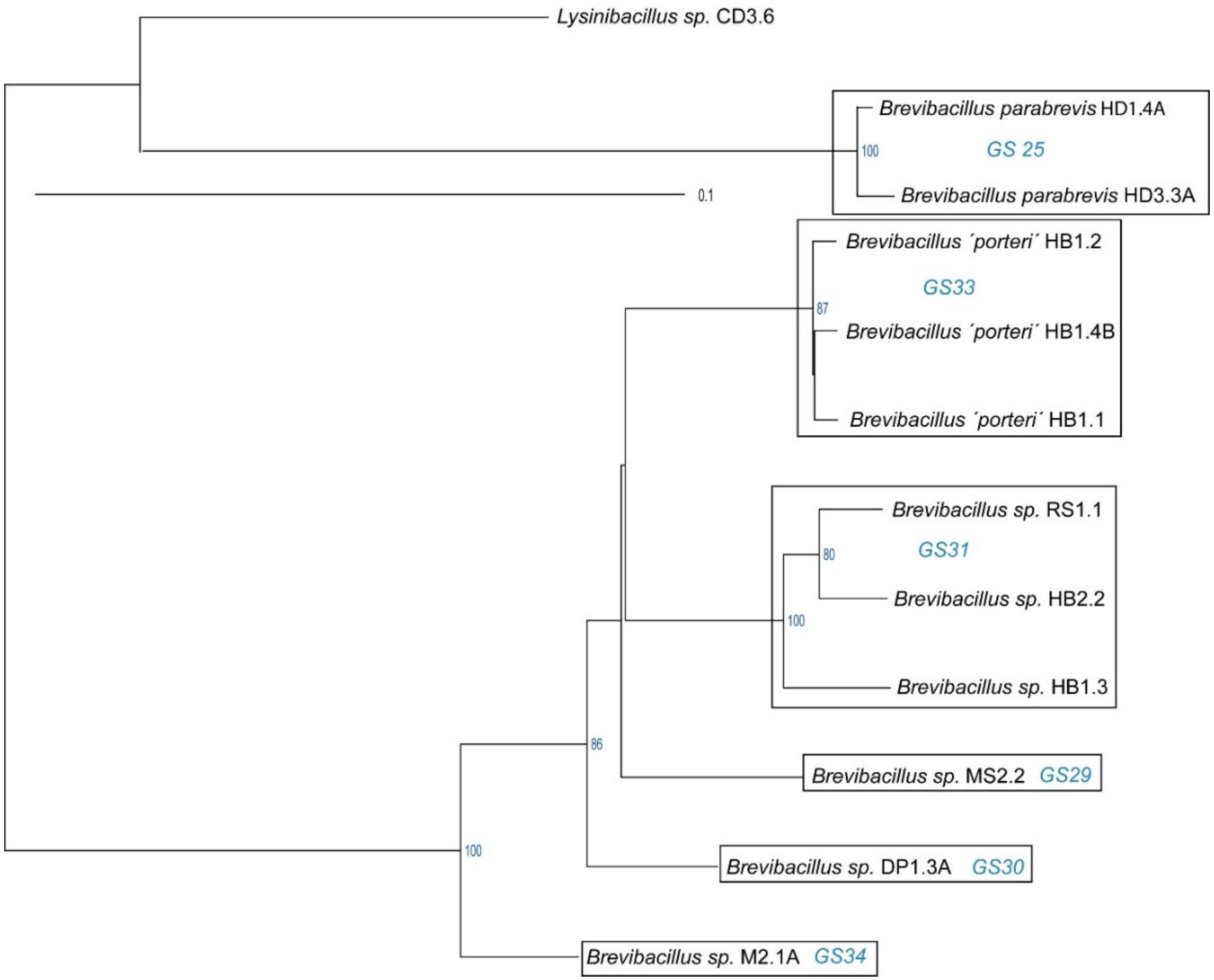


**Supplementary Figure S9**: Phylogenetic tree constructed with the Vietnamese *Brevibacillus* isolates. The corresponding genomospecies clusters are indicated. *Lysinibacillus* sp. CD3-6 was used as outgroup.

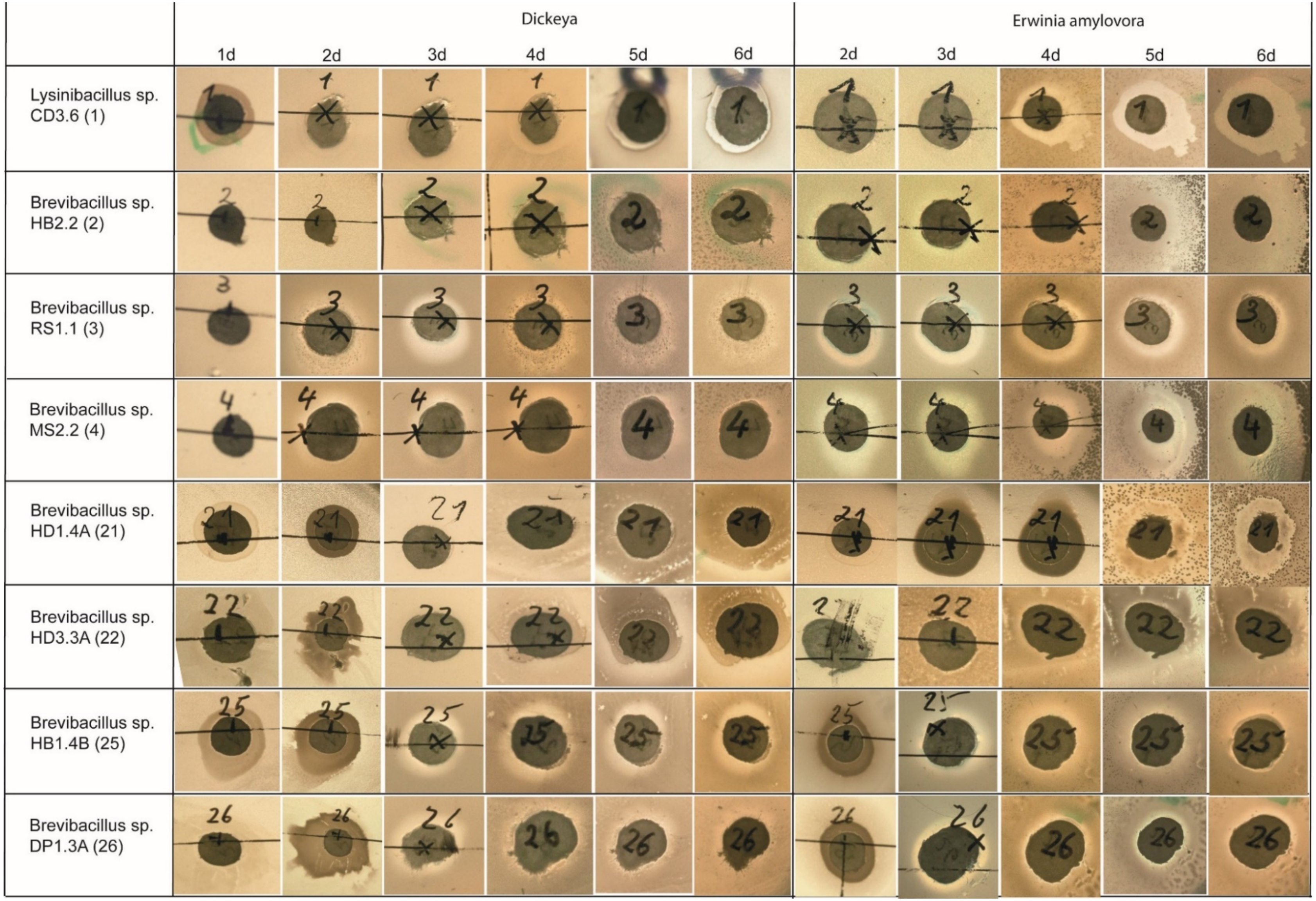


**Supplementary Figure S10**: In vitro assay of biococontrol action of *Lysinibacillus* and *Brevibacillus* strains against *Dickeya solani* and *Erwinia amylovora*.

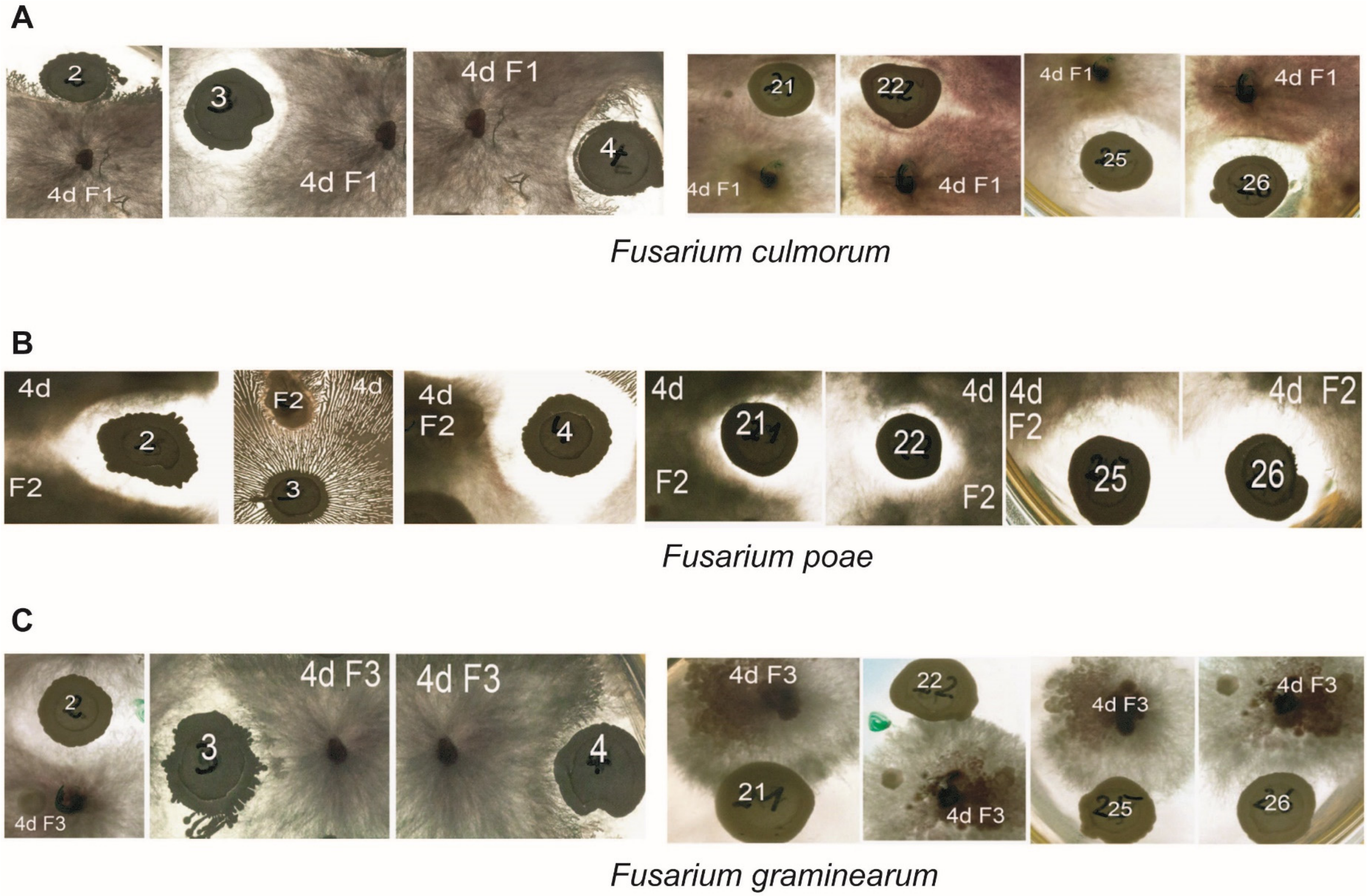


**Supplementary Figure S11**: Antagonistic action of *Brevibacillus* sp. HB2.2 (2), *Brevibacillus* sp. RS1.1 (3), *Brevibacillus* sp. MS2.2 (4), *B. parabrevis* HD1.4A (21), *Brevibacillus parabrevis* (22), *Brevibacillus porteri* HB1.4B (25), and *Brevibacillus* DP1.3A (26) against *Fusarium culmorum* (A), *Fusarium poae* (B), and *Fusarium graminearum* (C) after 4 days incubation at 27°C.

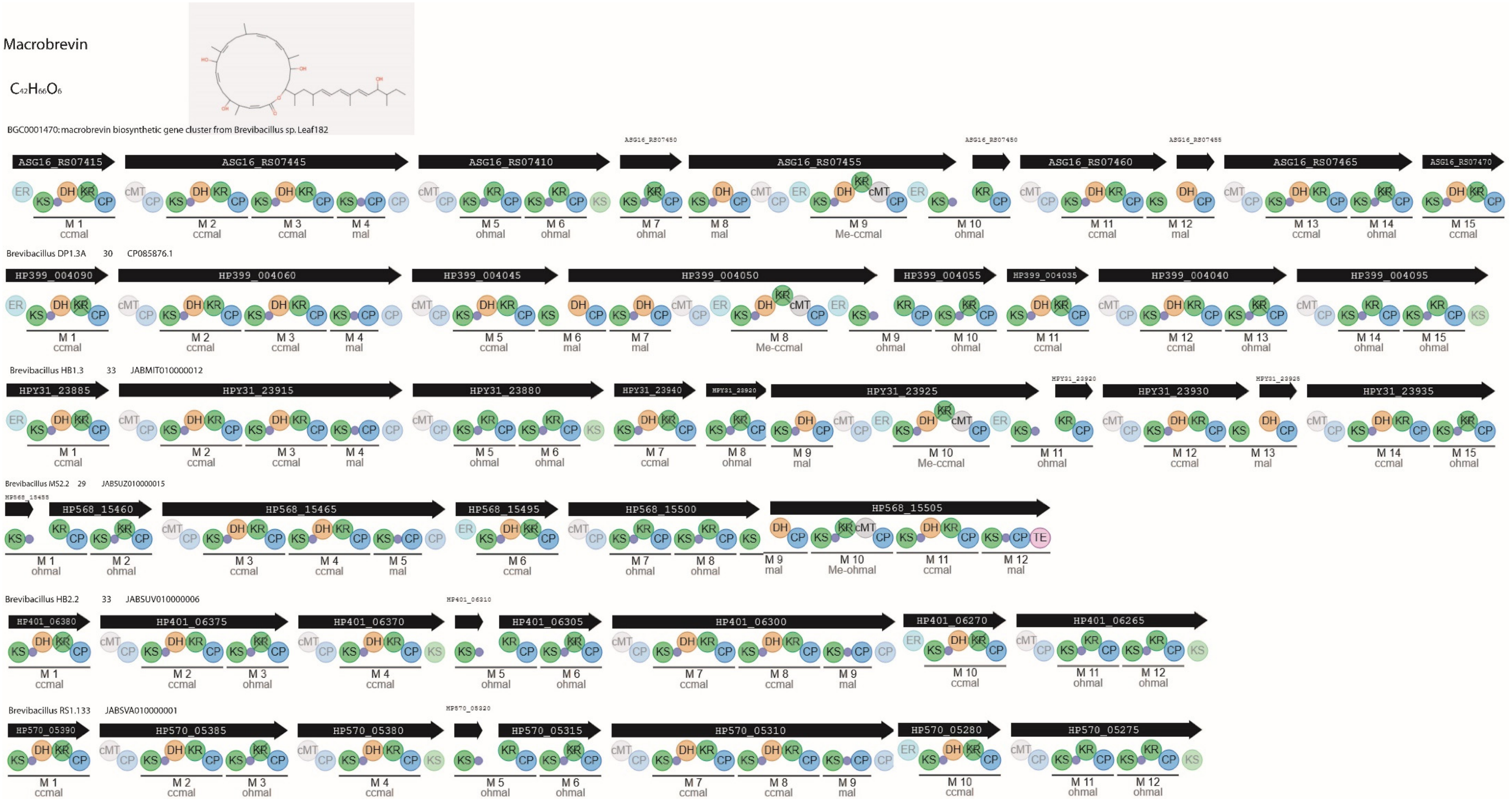


**Supplementary Figure S12:** Modular PKS in Brevibacilli: The Macrobrevin variants harbored slight differences compared to *Brevibacillus sp.* leaf 182 (BGC0001470). NRPS/PKS domains are indicated by filled circles. Fade circles indicate inactive domains. Crosses mean that the corresponding domain is not complete. CP: condensation domain, DH: dehydratase domain, KS: ketosynthase domain, KR: ketoreductase, TE: thioesterase.

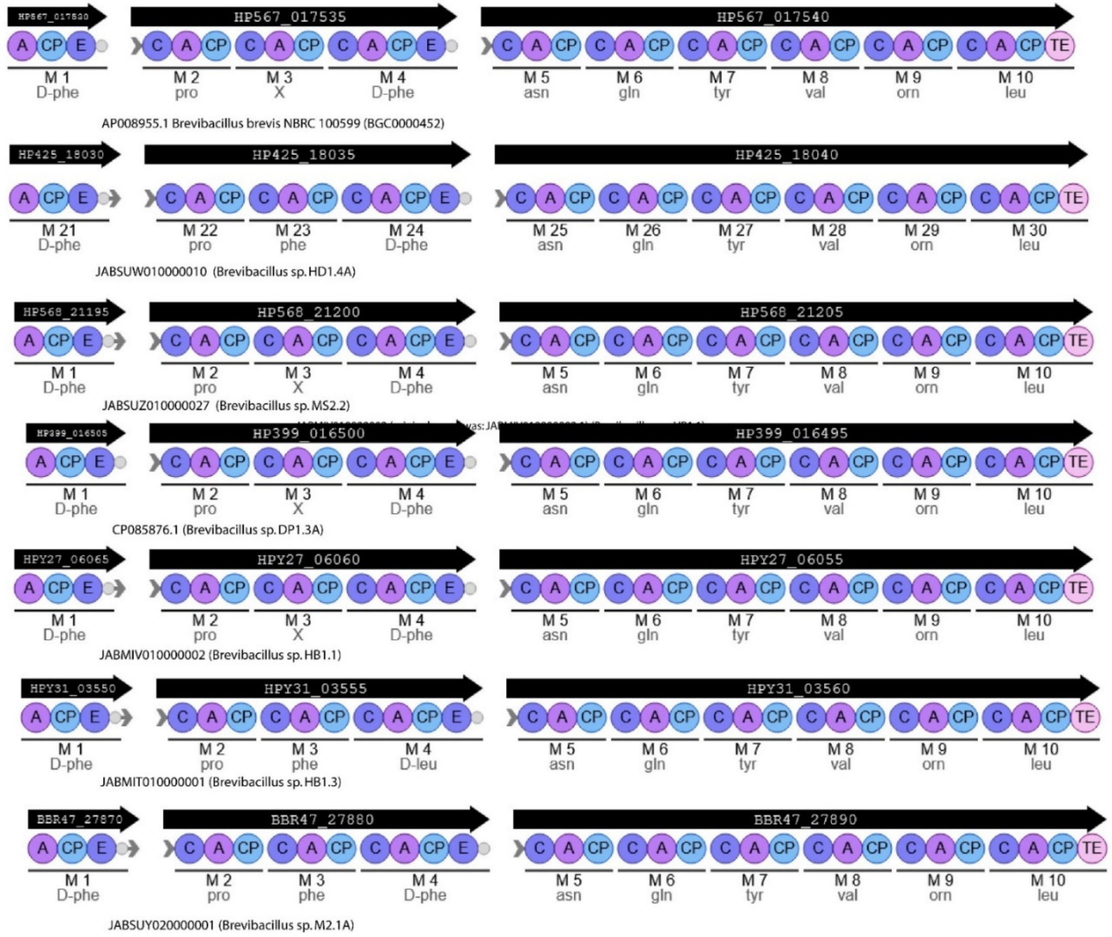


**Supplemental Figure S13:** NRP: Gene clusters similar to Tyrocidine (BGC0000452) present in *Brevibacillus parabrevis* HD1.4A (GS 25), *Brevibacillus sp.* MS2.2, DP1.3A, HB1.1, and M2.1A.

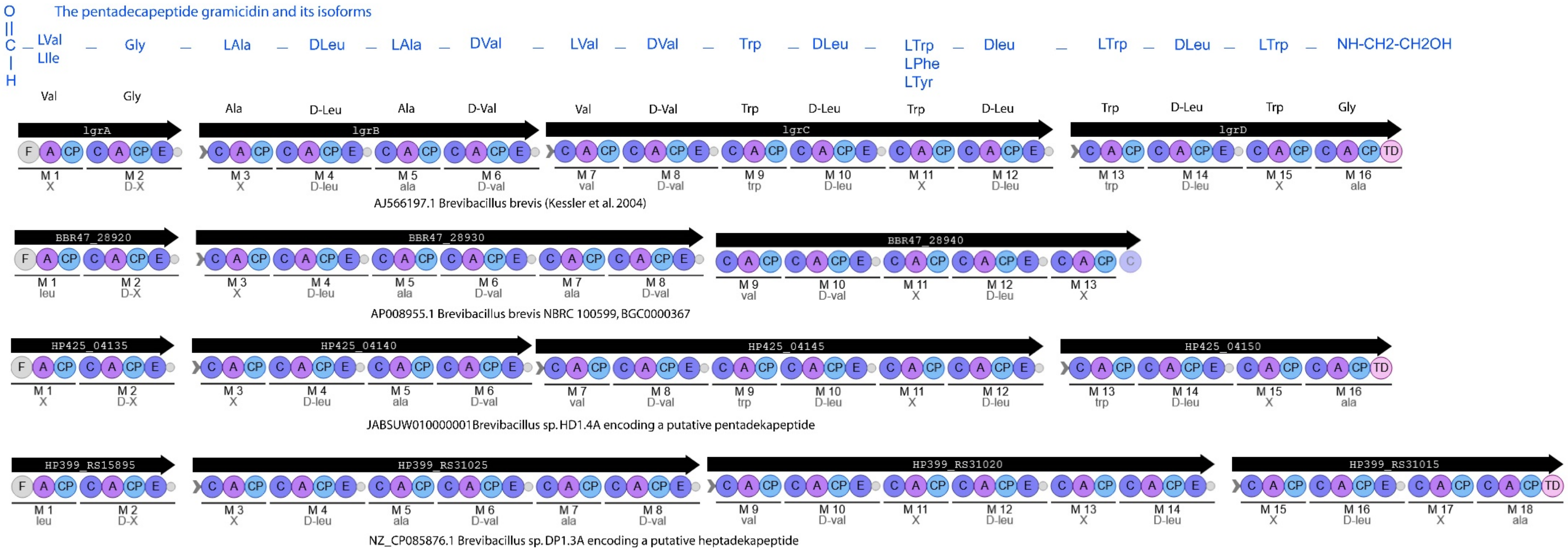


**Supplemental Figure S14:** NRP: Gene clusters similar to gramicidin (BGC0000367) and the gramicidin gene cluster in *Brevibacillus brevis* (AJ566197) were detected in *Brevibacillus parabrevis* HD1.4A (GS 25), and *Brevibacillus sp.* DP1.3A. The pentadecapeptides gramicidin A, B, C are shown above in blue letters. The complete gene clusters detected in *Brevibacillus brevis* (AJ566197), *Brevibacillus parabrevis* HD1.4A (JABSUW010000001), and *Brevibacillus sp.* DP1.3A (CP085876) were characterized by a formylation (F) domain in the first module (*lgrA*) and a final reductase (TD) domain in the last module (*lgrD*).

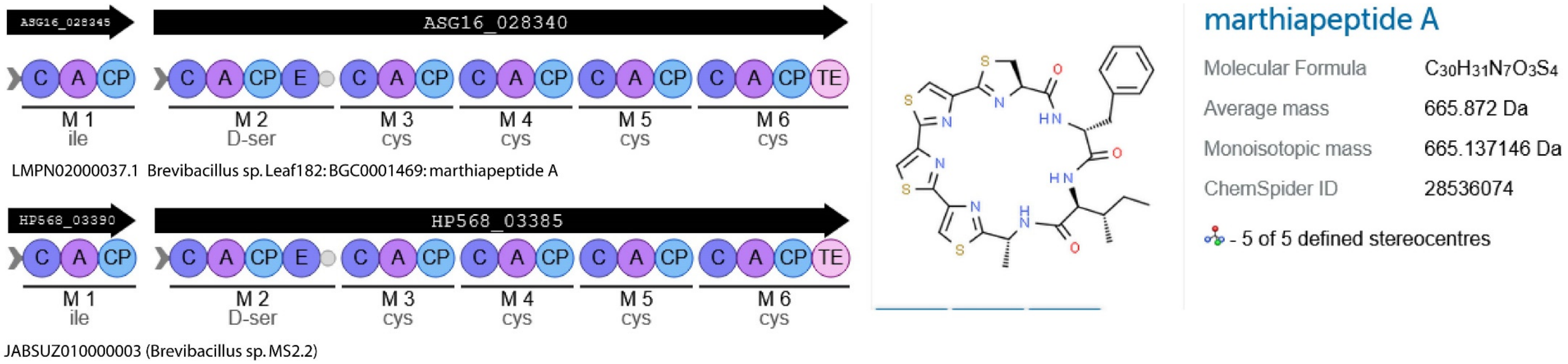


**Supplemental Figure S15:** NRP: Gene cluster similar to marthiapeptide A (BGC0001469) was detected in *Brevibacillus* sp. MS2.2. The structure of marthiapeptide A was taken from ChemSpider (ID 28536074)

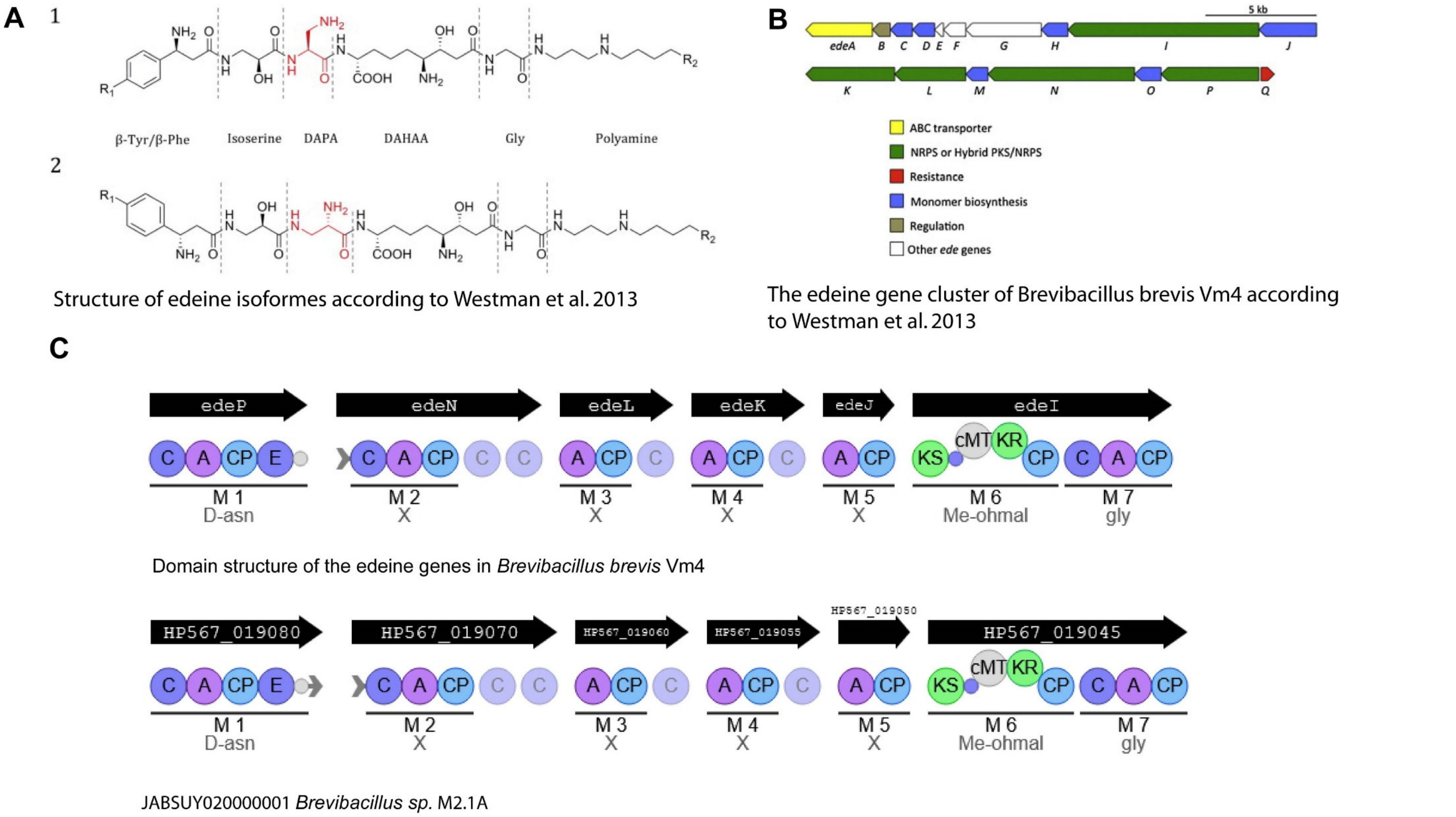


**Supplementary Figure S16:** Modular PKS-NRP hybrids. A: Structure of edeine isoforms according to Westman et al. 2013. B: The edeine gene cluster of *Brevibacillus brevis* Vm4. C: Prediction of the domain structure in the *Brevibacillus* M2.1A gene cluster similar to the edeine gene cluster of *Brevibacillus brevis* Vm4 in Vietnamese *Brevibacillus* genomes by **antiSMASH 6.0.**

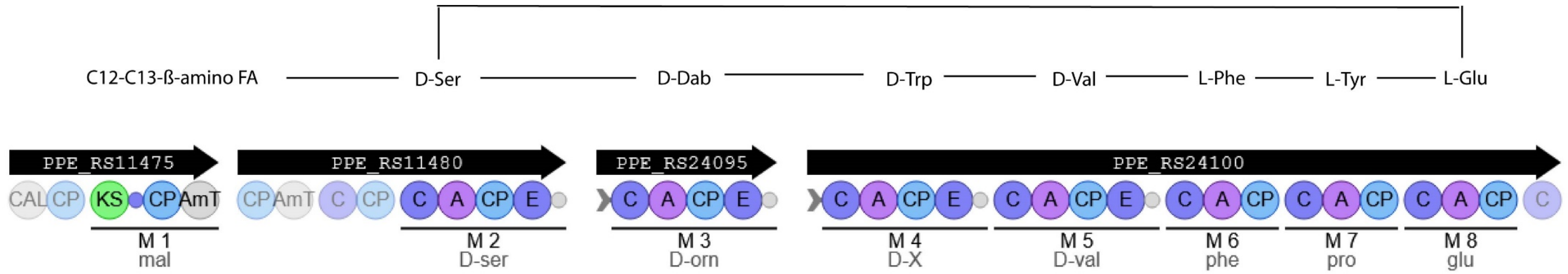


Domain structure of the Paenilipoheptin gene cluster of Paenibacillus polymyxa E681(BGC0001728) revealed by antiSMASH 6.0 analysis

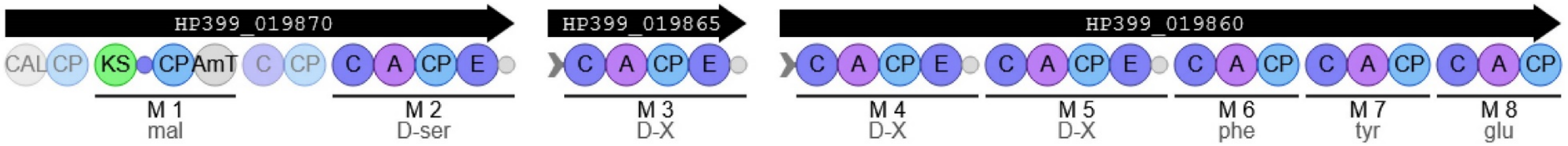


Domain structure of a gene cluster in Brevibacillus sp. DP1.3A similar to the Paenilipoheptin gene cluster of Paenibacillus polymyxa E681 (BGC0001728)

**Supplementary Figure S17:** Modular PKS-NRP hybrids. Gene cluster similar to the gene cluster encoding the synthesis of the cyclic lipoheptapeptide paenilipoheptin from *Paenibacillus polymyxa* E681 (BGC0001728) was detected in *Brevibacillus* sp.DP1.3A by antiSMASH 6.0 analysis. The sequence of paenilipoheptin is shown on the upper part (Vater et al. 2018).

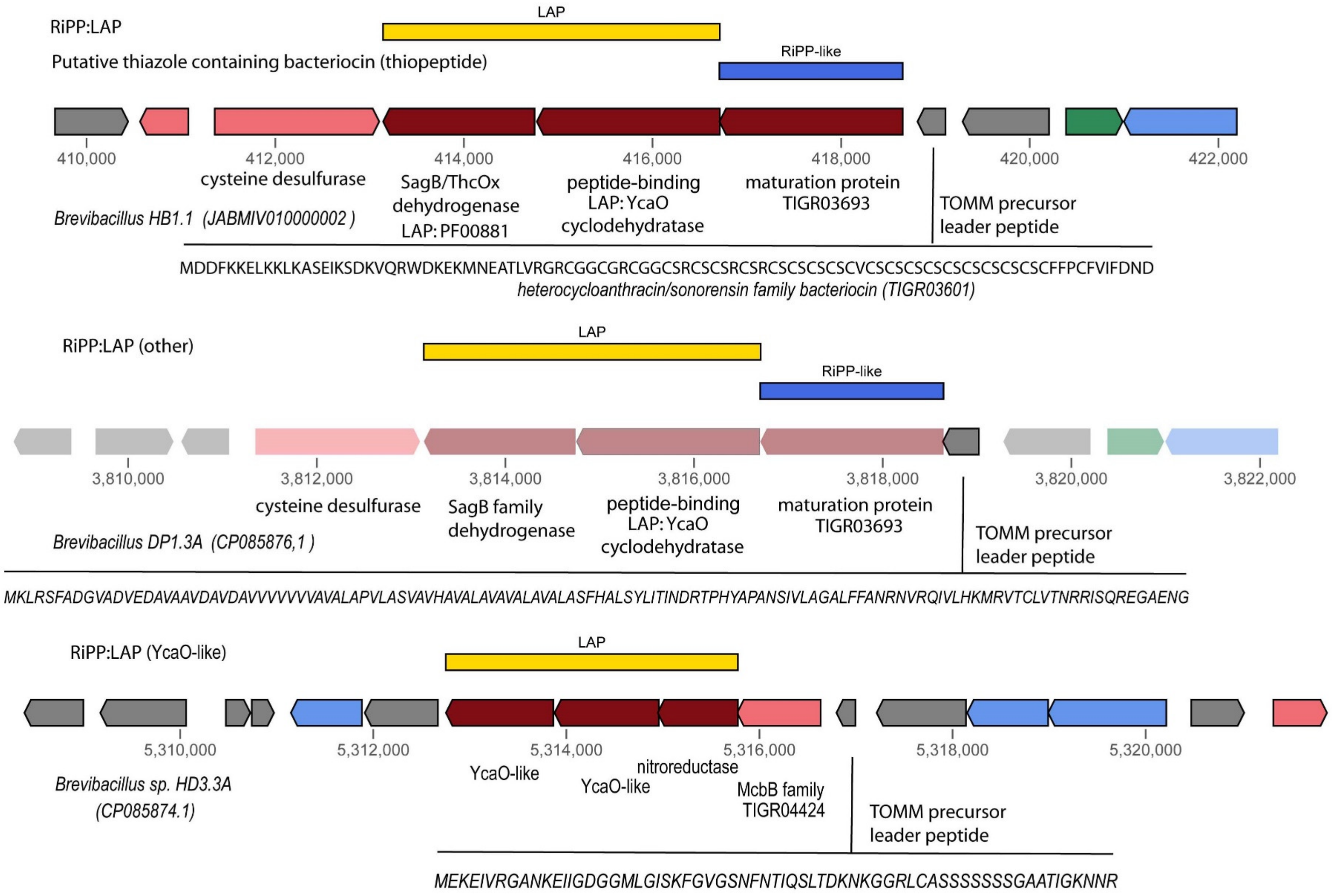


**Supplemental Figure S18:** RiPP-LAP in *Brevibacillus sp.* HB1.1, DP1.3A, andHD3.3A

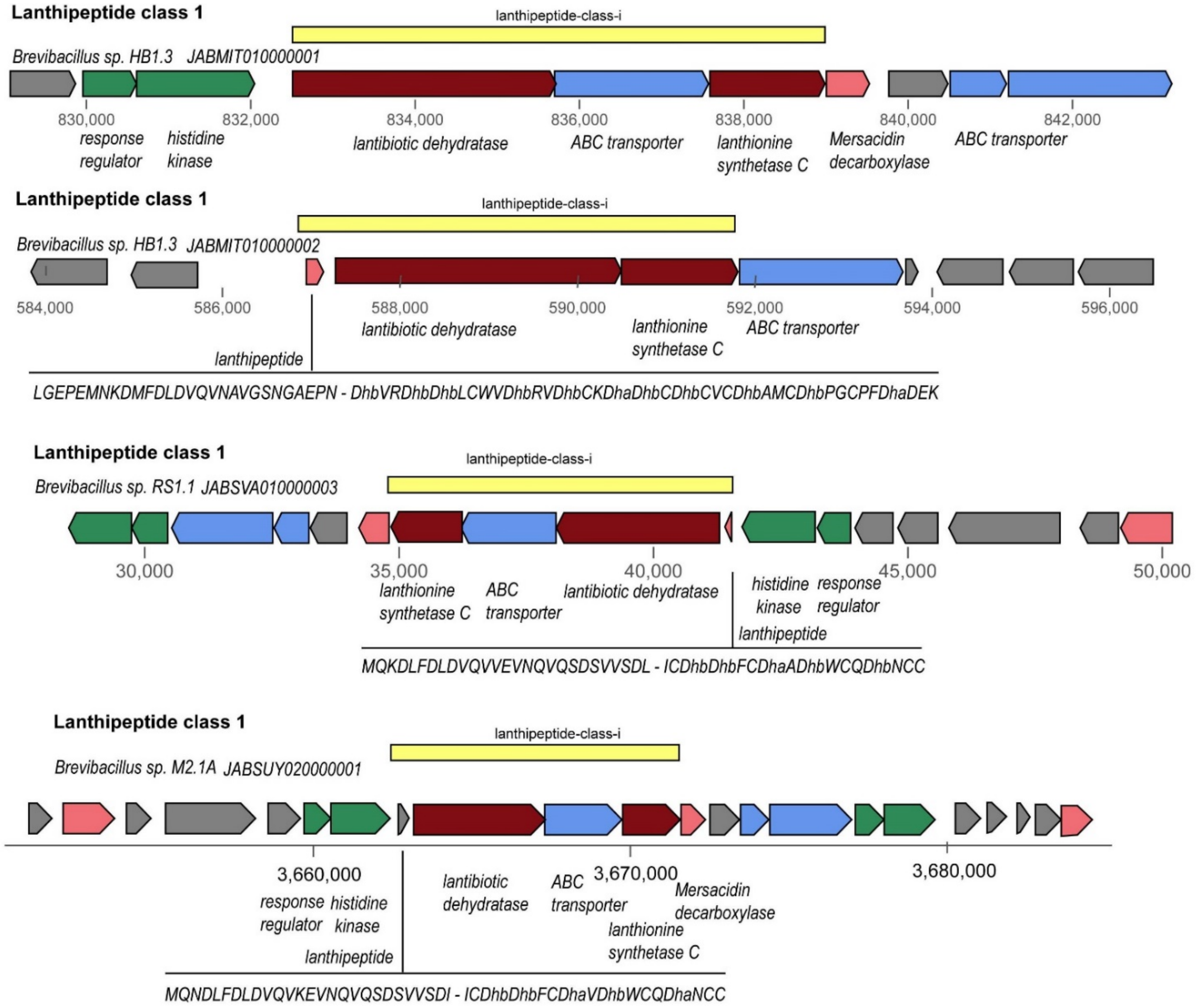


**Supplementary Figure S19:** Class 1 lanthipeptides detected in *Brevibacillus sp*. HB1.3, *Brevibacillus sp.* RS1.1, and *Brevibacillus sp.* M2.1A

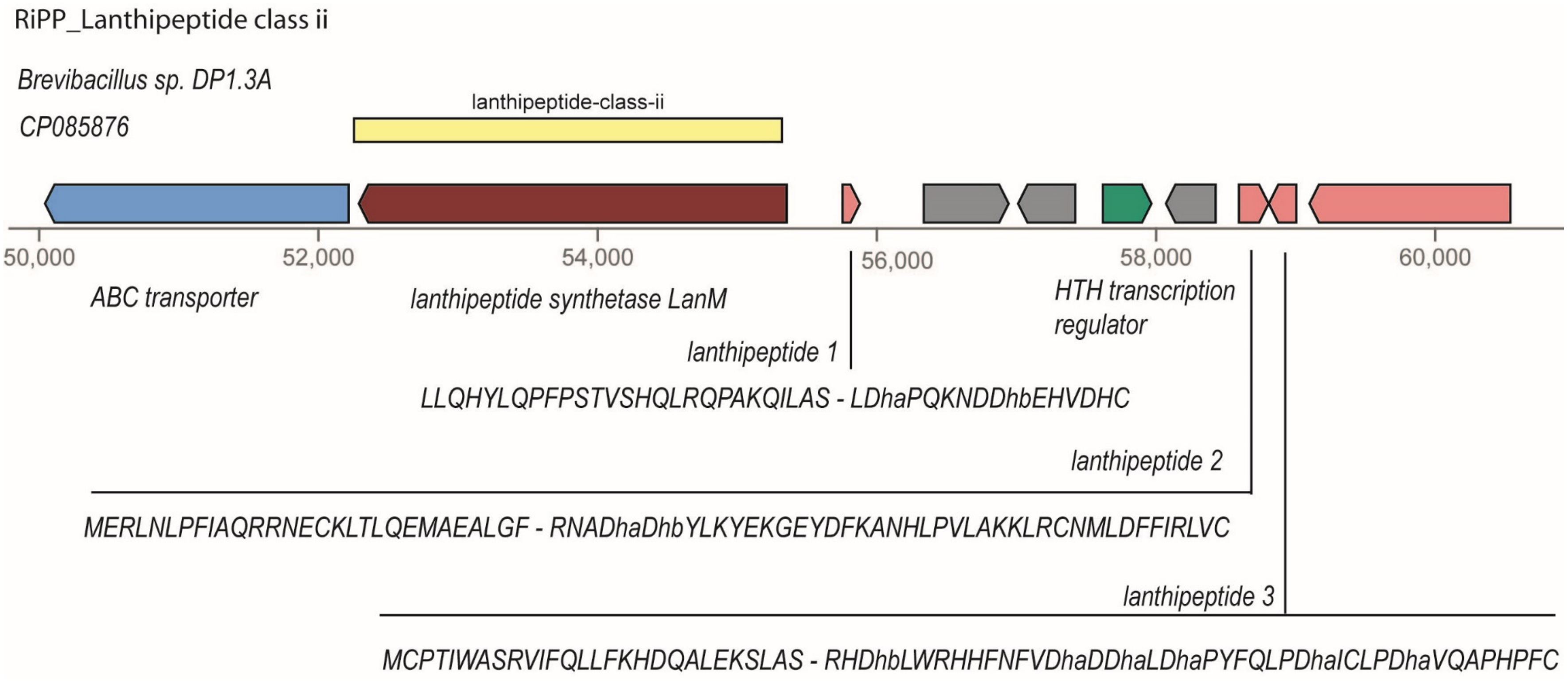


**Supplemental Figure S20:** RiPP-Lanthipeptides class 2 in *Brevibacillus sp.* DP1.3A

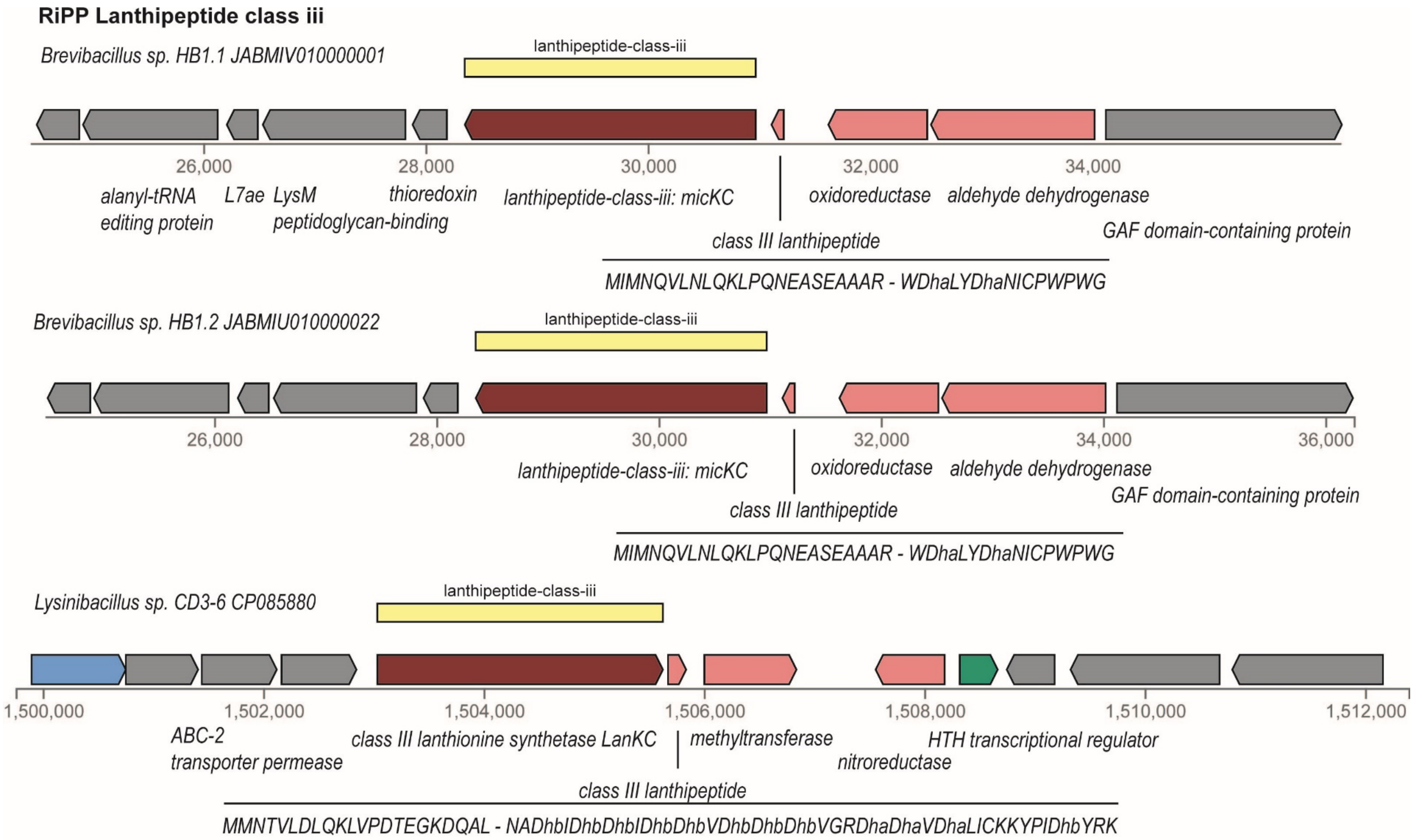


**Supplemental Figure S21:** RiPP lanthipeptides class 3 in *Brevibacillus porteri* HB1.1, HB1.2, and *Lysinibacillus* CD3-6

## UViB

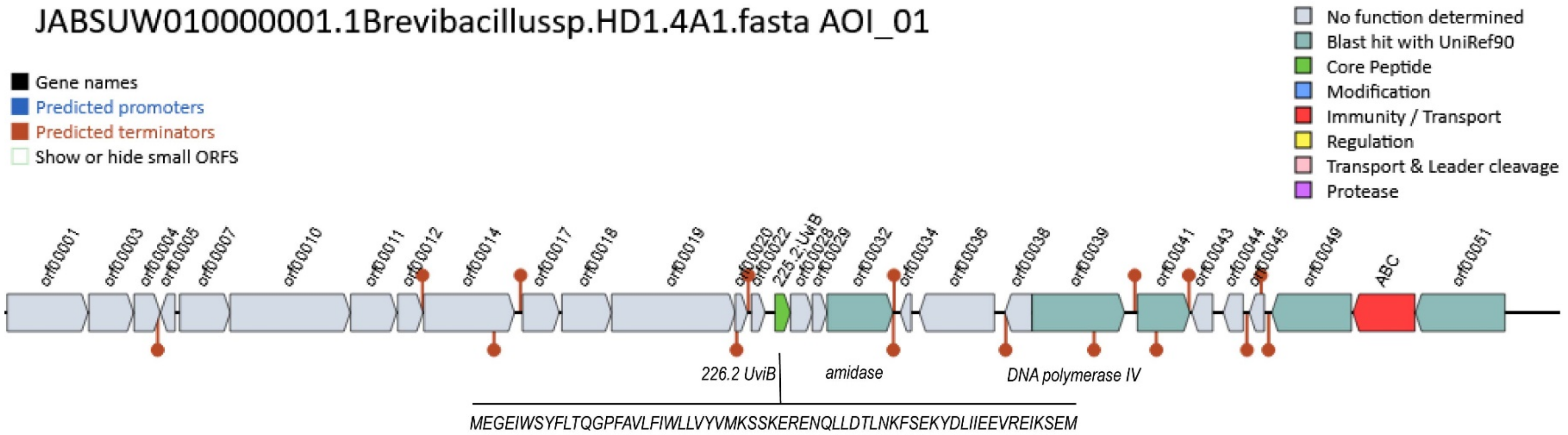


## Sactipeptide

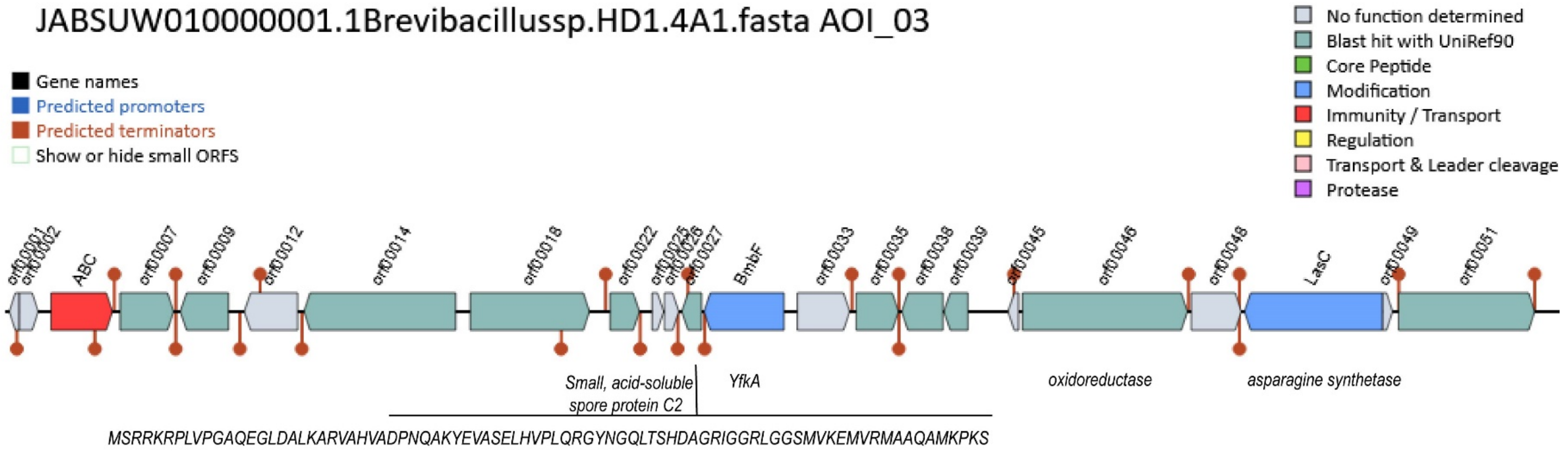


**Supplemental Figure S22:** UviB and sactipeptides were detected in Brevibacillus HD1.4A by BAGEL4

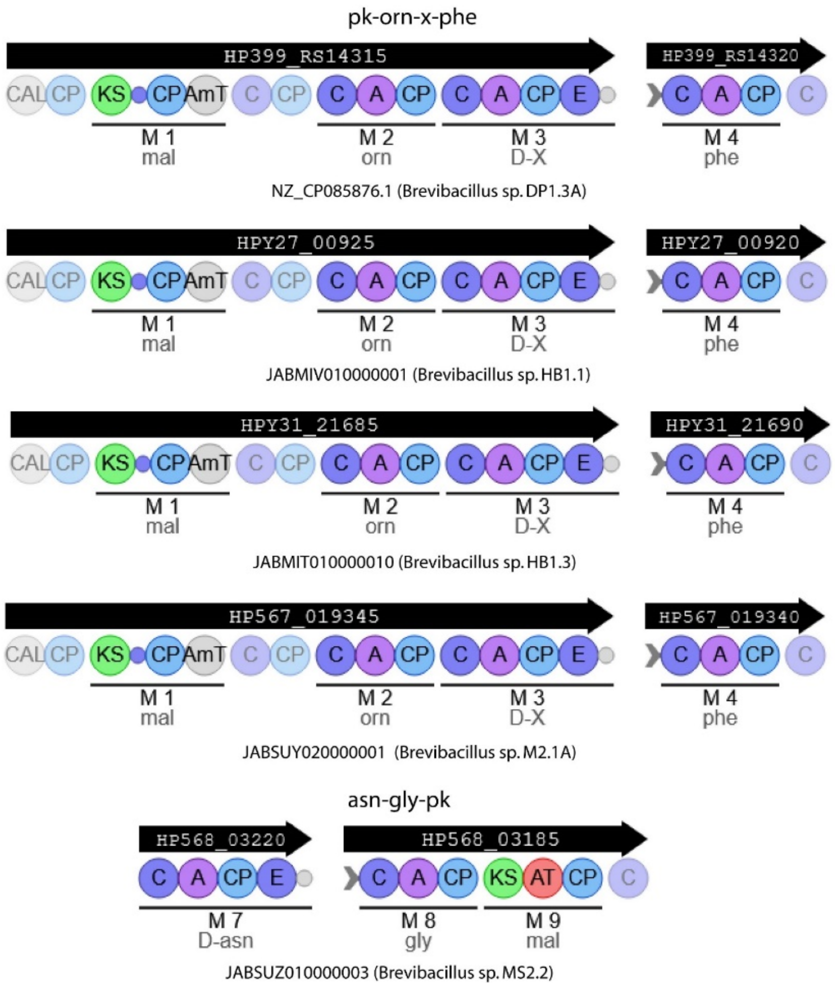


**Supplemental Figure S23:** Unknown PK-NRP hybrids pk-orn-x-phe and asn-gly-pk

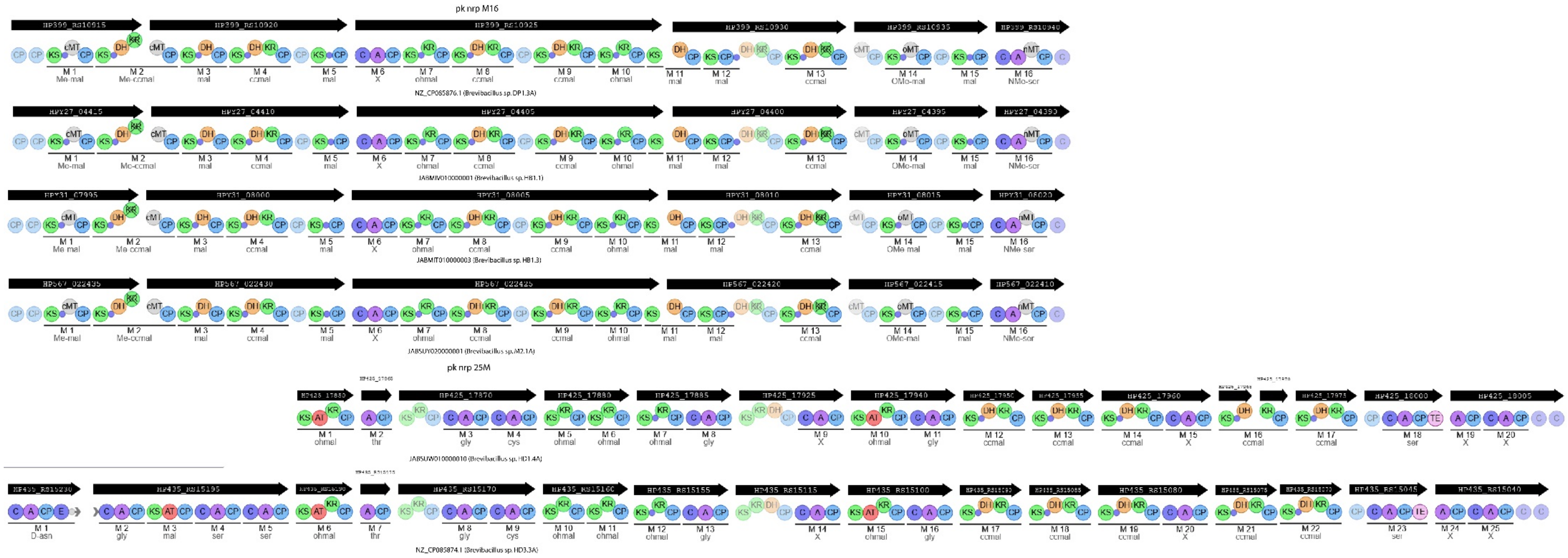


**Supplemental Figure S24:** Unknown PK-NRP hybrids: 16M PK-NRP and 20/25M PK-NRP

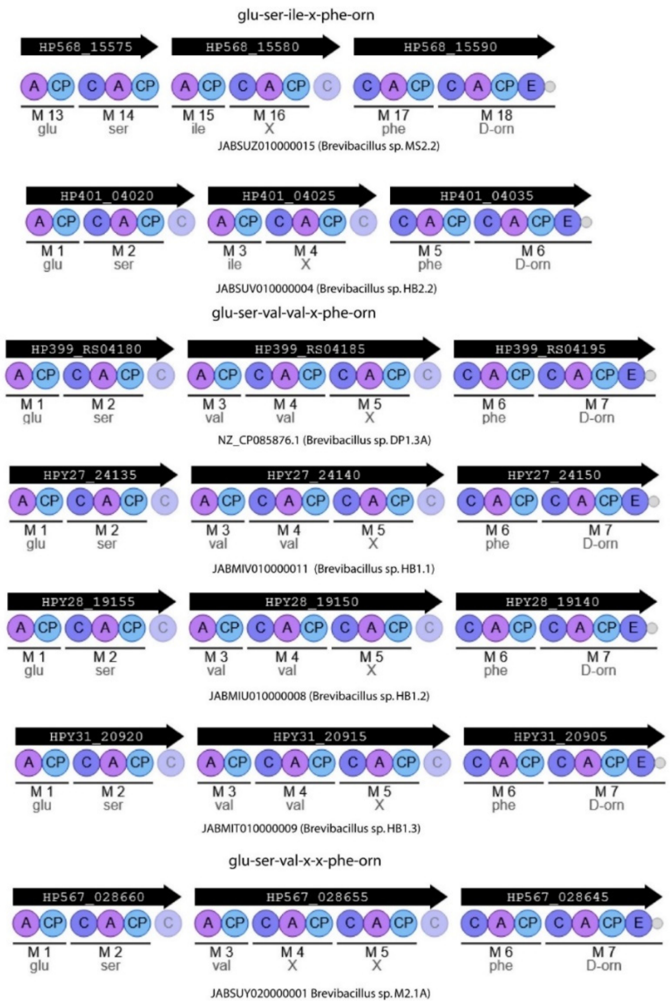


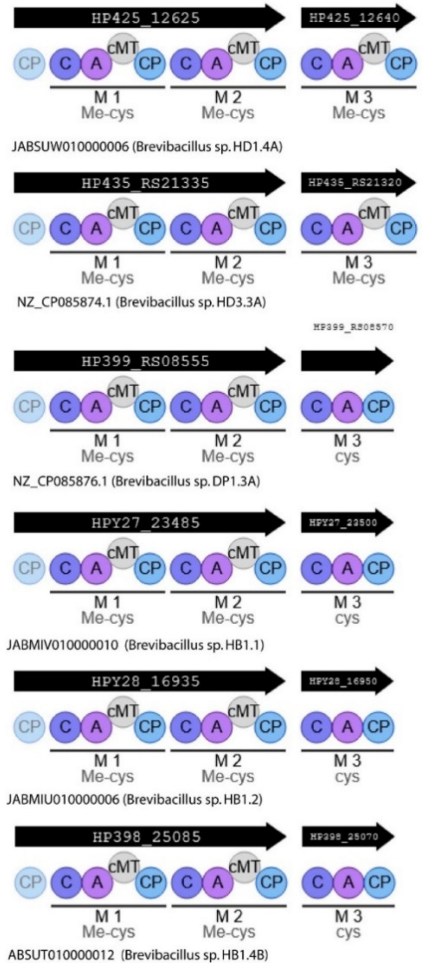


Left: **Supplemental Figure S25:** Unknown NRPS-like: glu-ser-ile-x-phe-orn/ glu-ser-val-val-x-phe-orn/ glu-ser-val-X-X-phe-D-orn (Brevipentin)

Right: **Supplemental Figure S26:** Unknown NRPS-like: cys-cys-cys (Brevitriazol)
